# Serum Concentration of Interleukin-6 Is Increased Both in Active and Remission Stages of Pemphigus Vulgaris

**DOI:** 10.1155/2008/875394

**Published:** 2008-06-23

**Authors:** Joanna Narbutt, Jolanta Lukamowicz, Jarosław Bogaczewicz, Anna Sysa-Jedrzejowska, Jolanta Dorota Torzecka, Aleksandra Lesiak

**Affiliations:** ^1^Department of Dermatology, Medical University of Lodz, 94-017 Lodz, Poland; ^2^Laboratory of Immunochemical Research, Polish Mother's Memorial Hospital Research Institute, 93-338 Lodz, Poland; ^3^Department of Immunodermatology, Medical University of Lodz, 94-017 Lodz, Poland

## Abstract

As most studies on pemphigus vulgaris (PV) pathogenesis concern its active stage, we aimed to evaluate the serum concentration of TNF-*α*, IL-1, and IL-6 in PV patients in clinical remission. The study group consisted of sera from 19 PV patients in active stage and from 24 patients in clinical remission. 19 sera taken from healthy subjects served as the controls. Serum IL-6 concentrations in PV active and PV remission group were significantly higher when compared to the controls (*P* < .05). In patients in active stage of PV, a significant correlation between serum IL-1 and IL-6 concentrations was found (*r*_*P*_ = 0.46; *P* < .05). We also found a negative correlation between TNF-*α* level and *pemphigus* antibodies titer in the patients from the remission group (*r*_*S*_ = −0.47303; *P* < .02). Our data suggest that IL-6 and TNF-*α* may be involved in maintaining immunological disturbances in remission stage of PV.

## 1. INTRODUCTION

Pemphigus is an autoimmune blistering disease, characterised by severe and chronic course. Based on clinical picture and profile of circulating *pemphigus* antibodies, two distinct forms of the disease are distinguished: pemphigus vulgaris (PV) and pemphigus foliaceus (PF). Transmembrane desmosomal proteins: desmoglein 1 (Dsg1) and desmoglein 3 (Dsg3) are pemphigus target antigens. In serum of PF patients, anti-Dsg1 antibodies are detected while in PV patients—antibodies are directed against Dsg3. However, in approximately 50% of the latter cases, also anti-Dsg1 antibodies may be found. Binding of *pemphigus* antibodies to the target antigens leads to the failure in adhesive function of desmogleins, and in consequence to disruption of desmosomes and thus acantholysis development [1]. Despite numerous investigations performed all over the world, the exact mechanism of acantholysis has not been fully elucidated. Recent studies point out at proinflammatory cytokines such as TNF-*α*, IL-1, or IL-6 as strong players involved in this process. Moreover, experimental studies revealed that synergistic cooperation of *pemphigus* antibodies with Fas-L and TNF-*α* results in acantholysis. Increased mRNA expression for TNF-*α* and enhanced cytokine's level in serum of PV patients were observed by many authors [2–4]. Furthermore, experimental models showed that mice lacking TNF-*α* receptor are less sensitive to pemphigus development after passive transfer of *pemphigus* antibodies [2]. The above data at least in part explain therapeutic efficacy of anti-TNF-alpha antibodies in pemphigus vulgaris treatment [5, 6].

Despite extensive research, the objective criteria of complete cure in PV have not been defined so far. Direct immunofluorescence (DIF) test detecting in vivo IgG deposits bound in the intercellular spaces of the epidermis and indirect immunofluorescence (IIF) test demonstrating circulating IgG antibodies are routine examinations in pemphigus diagnosis and their negative findings may indicate treatment cessation. In some cases, however, despite a long-lasting treatment and lack of clinical symptoms, either both immunological examinations are still positive, or DIF test is negative, but circulating antibodies are still detected. It seems controversial, especially because most dermatologists assume a strong correlation between the titre of *pemphigus* antibodies and the disease activity. Most studies on PV pathogenesis are focused on immunological disturbances in active stage of the disease.

Thus, the aim of our study was to evaluate the serum concentration of TNF-*α*, IL-1, and IL-6 in PV patients both in active stage of the disease and in clinical remission and assess their potential influence on disease course. We also assessed the correlation between cytokines' serum levels and titer of *pemphigu* s antibodies.

## 2. MATERIAL AND METHODS

The study group consisted of 19 sera obtained from PV patients in active stage of the disease and 24 sera obtained from patients in clinical remission. Sera from active PV patients had been collected before immunosuppressive treatment; (prednisone+cyclophosphamide) was introduced. 19 sera taken from healthy unrelated subjects, age and sex matched served as controls. Pemphigus vulgaris diagnosis was based on the clinical picture and results of histological and immunological examinations. The sera of all the patients and individuals from the control group were examined by IIF on commercially available substrate of monkey esophagus (Euroimmune, Lubeka, Germany), using a standard procedure [7]. The same sera samples were examined by ELISA (MESACUP Desmoglein Test Dsg1 and Dsg3; MBL Co. Ltd, Nagoya , Japan) according to the manufacturer's instruction. ELISA index was assessed as positive when it was higher than cutoff (for Dsg 1–14; for Dgs 3–7).

All the patients' direct immunofluorescence (DIF) tests revealed in vivo IgG and/or C3 deposits bound in the intercellular spaces of the epidermis. Active stage of the disease was considered when all the examinations were positive, blisters and erosions on the mucous membranes and/or skin were present. Sera samples were taken before introducing immunosuppressive treatment. Clinical remission was considered when patients were treated with prednisone (20–40 mg every other day) and cyclophosphamide (50 mg every other day) and demonstrated lack of clinical lesions for at least six months, while DIF and IIF still gave positive results.

The same sera samples were used to measure TNF*α*, IL-1, and IL-6 levels using commercially available ELISA kits (R & D, Minneapolis, Minn, USA), according to manufacturer's procedure.

## 3. STATISTICAL ANALYSIS

Statistical analysis was performed using Statistica v.0.5 and GraphPad Prism v.4.03. As laboratory data did not fit a Gaussian distribution according to Shapiro-Wilk test, all results were expressed as median (50th) and interquartile range (25th–75th), and nonparametric tests were used to test statistical significance. To avoid errors inherent in repeated application of Mann-Whitney U tests, the Kruskal-Wallis test was performed to make simultaneous comparison of the assay data from each group, and to determine whether there was a significant variation in the medians of the groups analysed. If this achieved 95% significance, Dunn's multiple comparison posttest was then used to compare the assay results of one group with the other. For correlation studies, the Pearson's correlation or Spearman's rank correlation was used. In all calculations, *P* < .05 was regarded as statistically significant.

## 4. RESULTS

Indirect immunofluorescence test revealed circulating *pemphigus* antibodies in all PV patients. In the subjects from active stage of the disease, antibodies titers ranged from 1:80 to 1:2560 (median 1:640) while in the patients from remission group from 1:20 to 1:1280 (median 1:80).

**Figure 1:**
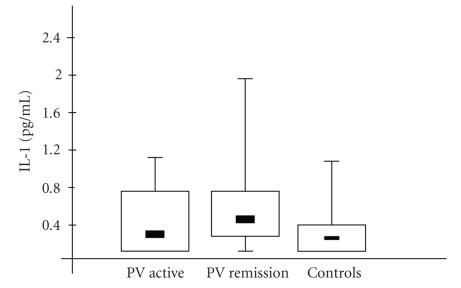
Interleukin-1 (IL-1) concentration in serum samples from patients with active stage of pemphigus vulgaris (PV active), remission stage of PV (PV remission), and in normal controls. Box plots with upper and lower bars showing the data range, and upper, middle, and lower lines in the box showing 75th, 50th (median), and 25th centiles, respectively.

**Figure 2:**
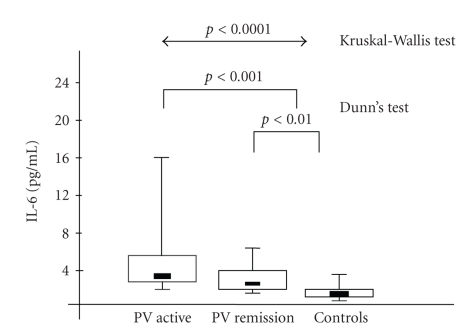
Interleukin-6 (IL-6) concentration in serum samples from patients with active stage of pemphigus vulgaris (PV active), remission stage of PV (PV remission), and in normal controls. Box plots with upper and lower bars showing the data range, and upper, middle, and lower lines in the box showing 75th, 50th (median), and 25th centiles, respectively. Kruskal-Wallis test across all three groups *P* < .0001. Only significant Dunn's multiple comparison posttest *p* values between groups are shown.

**Figure 3:**
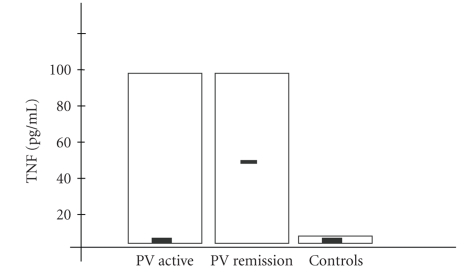
Tumor necrosis factor-*α* (TNF-*α*) concentration in serum samples from patients with active stage of pemphigus vulgaris (PV active), remission stage of PV (PV remission), and in normal controls. Box plots with upper and lower bars showing the data range, and upper, middle, and lower lines in the box showing 75th, 50th (median), and 25th centiles, respectively.

Median serum concentrations of IL-1, IL-6, and TNF-*α* in all the examined patients are presented in Table 1 and Figures 1–3. IL-1 serum concentrations in the PV patients from active and remission groups did not significantly differ from the controls. IL-1 concentration in the PV remission group was higher than in the PV active group, however, this did not reach statistical significance. Serum IL-6 concentrations in PV active and PV remission group were significantly higher when compared to the controls. IL-6 levels presented slightly higher values in the active group in comparison to the remission group, but the difference also did not reach statistical significance. Serum TNF-*α* levels did not differ significantly between PV active group, PV remission group and the control. The obtained values also did not differ between PV active and PV remission groups.

**Figure 4:**
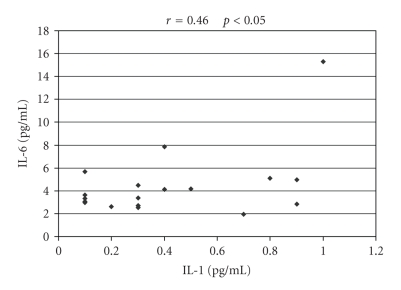
Correlation between serum concentrations of IL-1 and IL-6 in patients with active stage of PV.

A significant correlation between serum IL-1 and IL-6 concentrations in patients presenting active stage of PV was found (*r*_*p*_ = 0.46; *P* < .05) (see Figure 4). No similar relation was observed for the patients in the remission stage of PV. We also did not find any significant correlations between serum concentration of IL-1 and TNF-*α* or IL-6 and TNF-*α* in all the studied groups.

When analysing correlations between titer of circulating *pemphigus* antibodies and serum concentrations of the examined cytokines, we found a negative correlation between TNF-*α* level and antibodies titer in the patients from the remission group (*r*_*S*_ = −0.47303; *P* < .02).

## 5. DISCUSSION

Pemphigus vulgaris is a life-threatening disease of autoimmune background. During last decades, its pathogenesis has been widely investigated and new insight into mechanisms triggering disease development and production of antibodies has been recognised. Despite the fact of that detection of circulating and bound in vivo IgG *pemphigus* antibodies is that main immunological feature, current knowledge point out at involvement not only humoral but also cellular immunological response in pemphigus development. It was shown that Dsg3-specific lymphocytes T proliferate under desmoglein 3 stimulation release Th2-dependent cytokines such as IL-4 and IL-10 and demonstrate ability to modulate activity of B cells [8, 9]. Although it is obvious that autoimmune response in PV is T cell-mediated, the exact role of those cells in acantholysis development has not been elucidated yet. IL-2 is one of the main activators of T lymphocytes. Increased values of soluble receptor for IL-2 (sIL-2R) were detected in sera of patients with pemphigus, and these values correlated with activity of the disease. However, their concentrations were significantly higher in blister fluid thus suggesting presence of activated T cells in PV skin lesions [10]. It was also proven that isolated T lymphocytes from PV patients are required for induction of histological and immunological phenomena in the animal models of pemphigus [11]. Passive transfer of *pemphigus* antibodies into hairless mice genetically thymus deficient caused their binding in intercellular spaces of the epidermis, however blister formation over the basement layer of epidermis was observed in less than 20% of cases. It is another proof for an important role of lymphocytes T in acantholysis development [12].

Keratinocytes stimulated with serum obtained from PV patients presented higher expression of mRNA for IL-1*α*, TNF-*α*, and urokinase plasminogen activator (uPA) and developed acantholysis. When antibodies against these proteins were used in these experiments, the authors observed a significant inhibition of acantholysis [2, 13]. Alecu et al. [14] found increased levels of ICAM-1, TNF-*α*, and IL-6 in sera and blister fluid obtained from PV patients. The above results seem to at least in part confirm the role of these proteins in PV development. Similar results were obtained by D’Auria et al. and López-Robles et al. [3, 4] who showed increased serum levels and in situ expression of IL-6 and TNF-*α*. In experimental models, it was also demonstrated that IL-1*α* and TNF-*α* were able to activate C3 mRNA in keratinocytes culture after stimulation with *pemphigus* antibodies obtained from PV patients. This phenomenon is strongly inhibited after subsequent incubation of keratinocytes with anti-TNF-*α* and anti-IL1*α* antibodies [15].

D’Auria et al. [3] also showed a positive correlation between serum concentration of these cytokines and the number of skin lesions. In our study, we attempted to assess TNF-*α*, IL-1, and IL-6 sera concentrations in PV patients being either in the active stage of the disease or in clinical remission. In the patients presenting the active stage of the disease, only IL-6 serum concentration was significantly higher when compared to the control group. IL-1 and TNF-*α* serum levels did not differ between patients in the active stage and the controls. In the patients who were in clinical remission, we found similar results. Only IL-6 serum level was higher while the rest of the examined cytokines presented similar values to the controls. Decreased levels of IL-6 in remission stage of PV when compared to the active patients (statistically insignificant) may result from the immunosuppressive treatment administration. In many reports, it is noted that prednisone and cyclophosphamide influence proinflammatory cytokines levels, including IL-6 levels [16, 17]. Our results confirm the role of IL-6 in PV pathogenesis, not only in the initial stage of the disease development but also the pathophysiological process maintenance. In most published studies, the authors found an increased level of TNF-*α* in the active stage of pemphigus [4]. TNF-*α* is a cytokine involved in the majority of inflammatory processes, and its increased activity is found in many skin diseases including psoriasis, SLE, or systemic sclerosis [18–20]. TNF-*α* is released by cells under various stimuli including bacterial infections or ultraviolet radiation. It plays a role in many biological processes, enhances phagocytosis, cytotoxicity, and modulates activity of other cytokines such as IL-1 and IL-4 [21]. We did not confirm TNF-*α* increased concentration in PV patients when compared to the controls. However, its negative correlation with *pemphigus* antibodies titer and relatively increased values when compared to the controls (no statistical significance) may suggest its involvement in maintaining pathological mechanisms. Treatment with immunosuppressive agents is sufficient to reduce skin lesions formation and decrease antibodies production, however it probably does not influence disturbed cellular response. These data also partially explain why anti-TNF-*α* treatment is successful in refractory PV patients.

There is only a scarcity of data on cytokines' levels in sera of PV patients in clinical remission. Bhol et al. [22] revealed a marked reduction of IL-1*α* and IL-1*β* with simultaneously increased levels of IL-1R in sera of patients in clinical remission. Intravenous immunoglobulin (IVIG) therapy caused similar effects thus suggesting that its therapeutic effect could depend on modulation of IL1 isoforms and its receptor levels. Galle et al. [23] and Keskin et al. [17] revealed also that IVIG exerts anti-IL-6 properties thus to some extent, explaining mechanisms of its activity in PV patients. IL-10 concentration in serum was also significantly lower in patients in clinical remission when compared to the active ones and its values correlated with pemphigus antibodies titers [24]. To the best of our knowledge, there is no data on IL-6 serum concentration in the PV patients in clinical remission. Our data suggest the role of IL-6 in the immunological disturbances maintenance in remission stage of PV, namely, in the patients in whom, despite lack of clinical lesions, antibodies are still produced and possess the ability to bind to keratinocytes surface antigens. We believe that this observation suggests clinical application of anti-IL-6 antibodies in refractory cases of pemphigus vulgaris.

## Figures and Tables

**Table 1:** Interleukin-1 (IL-1), interleukin-6 (IL-6), and tumor necrosis factor-*α* (TNF-*α*) concentrations in serum samples from patients with active stage of pemphigus vulgaris (PV active), remission stage of PV (PV remission), and in normal controls.

Cytokines	PV active *N* = 19	PV remission *N* = 24	Controls *N* = 19
IL-1 (pg/mL)			
Median	0.3	0.5	0.3
(Interquartile range, 25th–75th)	0.1–0.7	0.3–0.7	0.1–0.4

IL-6 (pg/mL)			
Median	3.38	2.58	1.97
(Interquartile range, 25th–75th)	2.72–4.98	2.085–3.86	1.64–2.25

TNF-*α* (pg/mL)			
Median	6.2	53.05	3.9
(Interquartile range, 25th–75th)	4.1–100	2.8–100	3.4–5.6
